# Supplementary material for: Patterns of Intron Gain and Loss in Fungi
Source: PLoS Biol. 2004 Nov 30;2(12):e422. doi: 10.1371/journal.pbio.0020422 (PMC532390; doi:10.1371/journal.pbio.0020422)
Supplement: Table S1 — Also available at http://genes.mit.edu/NielsenEtAl/. (4.3 MB ZIP). [file pbio.0020422.st001.zip › NielsenEtAl/html/121.html]

AN4428.1.NCU01481.1.MG07178.1.FG04398.1


```
 CLUSTAL W (1.82) Multiple Sequence Alignments - Introns Inserted


Sequence 1: NCU01481.1	623 aa
Sequence 2: FG04398.1	553 aa
Sequence 3: MG07178.1	645 aa
Sequence 4: AN4428.1	555 aa
Alignment Length: 675 aa
Number Identitical Residues: 247 aa
Alignment Score (without introns) 11462


MG07178.1 	MAKARSSFLGSS--SSTPR-----ANDNDEDVGLLASHN------QDVAHAAAAITSPRS
NCU01481.1	MGKAAHESYGAGAGAGGPRTASTHKNHNDENVGLLSGGSPNLLDDDGYDGDSTTLTGNGV
FG04398.1 	MTHARNN--------GSP-------NPHGEDRGLLSHGP-------DYDDDYFGIVADDH
AN4428.1  	MPGDERQ-----------------QHSQAETNGHAEVES-------QHLLSDESKKENND
          	*     .                  : : *  *                           

MG07178.1 	VPGDPEQQQQQQHQQTHQQTSDSK--------PPSRRPQSLQNTERRPSALHSPSLTGES
NCU01481.1	GAGPGPGTGTAGEQREEGSNTSPPVVDPRPAPAPAPVPAPAPGHIKLPRRQSALSAPRTP
FG04398.1 	-SGSGSGSASADLGKASGKTDSN-------------------------------------
AN4428.1  	TLHPNISSSVPDEPPSRPTSFSPP--------------------------------DRER
          	                   . .                                      

MG07178.1 	RTPRTPNRVRFDLQPIVVGGGVDAATANNGRRTSNGSAPGGPGQGLGGEFNHPLLALSSP
NCU01481.1	RTPRTPNRVRFDLRPTTFGDNSPTTTAPNG-------------NNGGAPEDHHHHARDSF
FG04398.1 	GQPRTPGRVRFDLTP-------EIVPVSNG-------------QSFGGFRDDDSERRSSD
AN4428.1  	RPPRTQNRVRFDIAD-----DSEEESRDNG-----------------VNRDSDDVLWLDE
          	  *** .*****:       .    .  **                    :       . 

MG07178.1 	SSRSSFDSYDSEFGVDSEPENAETERRLPLLTNIEAPSVSLANSGAEWANGG-AEWAED-
NCU01481.1	DSYFDIDDSSSDQQPLTSFSDYHRQGRVPLLTGIEAPSVTVANNGPWGSDEDVASWAETE
FG04398.1 	DAYFDIEEATSP----------ARAHRTPLLTDMEAPSVTLAN-----SMGDPSELAEHE
AN4428.1  	EDYVDRGRSGRH----------HAGQRIPLLTNIEAPSVTLAT------SEEFFPEEHLE
          	.   .                     * ****.:*****::*.              . .

MG07178.1 	HNRPKSGLRAAFMNMANSIIGAGIIGQPYAFKNAGLLAGVFLLISLTVVVDWTICLIVIN
NCU01481.1	RSRPKSGLRMAFMNMANSIIGAGIIGQPYAFKQAGLLSGIVLLVGLTVVVDWTIRLIVIN
FG04398.1 	MNRPKSGLKSAFMNMANSIIGAGIIGQPYAVRQAGLVGGILLLVGLTVVVDWTICLIVIN
AN4428.1  	SARPRSGMRMAFMNMANSIIGAGIIGQPYALRQSGMVTGILLLVALTVTVDWTIRLIVVN
          	  **:**:: ********************.:::*:: *:.**:.***.***** ***:*

MG07178.1 	SKLSGANSFQGTVERCFGKPGLIAVSVAQWAFAFGGMVAFGVIVGDSIPHVFLAIWPDLR
NCU01481.1	SKLSGANSFQGTVEKCFGRSGLIAISVAQWAFAFGGMVAFGVIVGDSIPNVLRAIWPGLR
FG04398.1 	SKLSGTSHFQGTVKHCFGQSGLIAISVAQWVFAFGGMVAYGVIVGDTIPHVLVAVWPNLS
AN4428.1  	SKLSGADSFQATMQHCFGKSGLIAISVAQWAFAFGGMIAFCIIVGDTIPHVLSSLFPSLR
          	*****:. **.*:::***:.****:*****.******:*: :****:**:*: :::*.* 

MG07178.1 	----NIPVLGLLADRRAVIVIFTLGISYPLTLYRDISK0LAKASTFALVSMLVIVFTVVI
NCU01481.1	EEGVEGTLVGWLVGRQGVILVCTLGVSYPLALYRDIAM0LAKASTLALVSMAVILVTVLV
FG04398.1 	----EVPVIGLLANRQVAIAVFVLGIGYPLTLYRDISK0LAKASTFALVGMVVIVFTILV
AN4428.1  	----EMSFLWLLTDRRAIIVLLVLGISYPLSLYRDIAK0LAKASTLALVSMAVIVIAVVT
          	    : ..:  *..*:  * : .**:.***:*****:  ******:***.* **:.::: 

MG07178.1 	QGLLTPKELRGSFDTS-LLTVNTGIAQAIGVISFA1FVCH~HNSLLIYGSLEKPTIDRFS
NCU01481.1	QGGLAPEEDKGTLASWNLLIINDGIFQAIGVISFA1FVCH~HNSLLIYGSLKTPTIDRFS
FG04398.1 	QGIVAPASERGSFSPS-LLLFNGGFFQAIGVISFA1FVCH~HNSLLIYGSLKTPTIDNFS
AN4428.1  	QGFRVPQDSRG--DVKNLLLLNTGFFQAVGVISFA1FVCH1HNSLLIYGSLKKPTLDRFA
          	**  .* . :*     .** .* *: **:****** **** **********:.**:*.*:

MG07178.1 	RVTHYSTGVSMLACLLMALAGFLTFGDKTLGNVLNNFPADNTMVTIARL~CFGLNMLTTL
NCU01481.1	LVTHISTGVSMIACLLMALVGFLTFGDRTLGNVLNNFPADNTMVNVARL~CFGLNMLTTL
FG04398.1 	RVTHYSTGVSMVFCLVLALGGFLTFGDKTMGNVLNNFPADNTMVNIARL~CFGLNMLTTL
AN4428.1  	KVTHYSTGISLLMCLLMGVSGFLFFGSETQGNVLNNFPSDNILINIARL2CFGLNMLTTL
          	 *** ***:*:: **::.: *** **..* ********:** ::.:*** **********

MG07178.1 	PLEAFVCREVMFNYYFPGDPFNLNLHLIFSSALVVSAMALSLMTCDLGVIFELVGSTSAC
NCU01481.1	PLEAFVCREVMLNYWFPGDPFNMNLHLLFTSSLVVSAMVLSMITCDLGTVFELVGATSAA
FG04398.1 	PLEAFVCREVMLTYFFPDEPFNMNRHLLFSTSLVVSALVLSLVTCDLGAVFELVGATSAV
AN4428.1  	PLEAFVCREVMTTYYFPDEPFNMNRHLIFTSALVLTSVAMALLTCDLGAVFELIGATSAA
          	*********** .*:**.:***:* **:*:::**::::.::::*****.:***:*:*** 

MG07178.1 	AMAYILPPLCYIKLASRS--WRTYCAWAVVVFGCLVFVVS~LFQSLEKIITG1NQAVLVV
NCU01481.1	AMAYILPPLCYLKLTKETGGWKRAAAWATAAFGTLVMGIM1-----------~-------
FG04398.1 	AMAYILPPLCYIKLTTRS--WRTYMAGAVVAFGIVVMTIS~VIQAVQKMVNS1KDGPAQC
AN4428.1  	SLAYIFPPLCYIKLSNGSQ-KAKIPAYACIVFGVTVMGVS~LLQAVGKMIKN1EGGTATC
          	::***:*****:**:. :       * *  .**  *: :    .:  .  .. . .    

MG07178.1 	HRGLDTTEHSCLLWYFMPAST
NCU01481.1	---------------------
FG04398.1 	A--------------------
AN4428.1  	ST-------------------
          	
```
